# ALPPS (associating liver partition and portal vein ligation for staged hepatectomy) does not affect proliferation, apoptosis, or angiogenesis as compared to standard liver resection for colorectal liver metastases

**DOI:** 10.1186/s12957-017-1121-8

**Published:** 2017-03-07

**Authors:** Katharina Joechle, Christian Moser, Petra Ruemmele, Katharina M. Schmidt, Jens M. Werner, Edward K. Geissler, Hans J. Schlitt, Sven A. Lang

**Affiliations:** 1 Department of Surgery, University Hospital Regensburg, Franz-Josef-Strauss Allee 11, 93053 Regensburg, Germany; 2 Department of Pathology, University Hospital Regensburg, Franz-Josef-Strauss Allee 11, 93053 Regensburg, Germany

**Keywords:** Associating liver partition and portal vein ligation for staged hepatectomy (ALPPS), Colorectal liver metastases (CRLM), Apoptosis, Proliferation, Vascularization

## Abstract

**Background:**

ALPPS (associating liver partition and portal vein ligation for staged hepatectomy) is a novel two-stage strategy to induce rapid hypertrophy of the future liver remnant (FLR) when patients are in danger of postoperative liver failure due to insufficient FLR. However, the effects of ALPPS on colorectal liver metastases (CRLM) are not clear so far. The aim of our study was to determine whether ALPPS induces proliferation, apoptosis, or vascularization compared to standard (one-stage) liver resection.

**Methods:**

Six patients who underwent ALPPS were matched with 12 patients undergoing standard liver resection regarding characteristics of the metastases (size, number), time of appearance (syn-/metachronous), preoperative chemotherapy, primary tumor (localization, TNM stage, grading), and patient variables (gender, age). The largest resected metastasis was used for the analyses. Tissue was stained for tumor cell proliferation (Ki67), apoptosis (TUNEL, caspase-3), vascularization (CD31), and pericytes (αSMA).

**Results:**

Vascularization (CD31; *p* = 0.149), proliferation (Mib-1; *p* = 0.244), and αSMA expression (*p* = 0.205) did not significantly differ between the two groups, although a trend towards less proliferation and αSMA expression was observed in patients undergoing ALPPS. Concerning apoptosis, caspase-3 staining showed significantly fewer apoptotic cells upon ALPPS (*p <* 0.0001), but this was not confirmed by TUNEL staining (*p =* 0.7344).

**Conclusions:**

ALPPS does not induce proliferation, apoptosis, or vascularization of CRLM when compared to standard liver resection.

## Background

In colorectal liver metastases (CRLM) and primary liver cancer, surgery offers the only chance for cure [1–4]. Nonetheless, 70 to 80% of patients suffering from CRLM are regarded to be irresectable upon presentation. However, the terms “resectability” and “irresectability” in liver surgery are not well defined, although several attempts have been made to do this over the last decades [5–7]. One of the major problems that limit especially extended liver surgery is the postoperatively remaining liver volume, also named future liver remnant (FLR). So far, approximately 20–25% of well-perfused normal tissue is regarded to be sufficient to maintain the postoperative liver function in otherwise healthy livers [8, 9]. Another option to calculate the needed FLR is to refer the volume of the FLR to the body weight (BW). A FLR/BW ratio of more than 0.5 is suggested to be enough to prevent posthepatectomy liver failure (PHLF) [10].

To address the issue of small FLR, several strategies to increase the remaining liver have been developed over the last years. In particular, two-stage hepatectomy (TSH) with or without interstage chemotherapy, as well as portal vein embolization (PVE) of the contralateral lobe, is currently in use [11–13]. Waiting time between the two procedures is 8–12 weeks and 4–6 weeks after TSH and PVE, respectively [14]. Although significant improvement of resectability has been achieved with these techniques, a number of patients remain irresectable either because of an inappropriate increase in FLR or because of tumor progression during the waiting period until resection [13, 15, 16]. We have recently introduced a novel two-stage approach for rapid hypertrophy of the FLR in extended right hepatectomy by combining a complete transection of the liver along the falciforme ligament with dissection of the right portal vein, known as ALPPS (associating liver partition and portal vein ligation for staged hepatectomy) or “in situ split liver resection” (ISS) [17]. After a median of 9 days, an increase in FLR (Seg. II/III) of 86% was observed, allowing rapid resection of the tumor-bearing liver lobe [18]. Moreover, the feasibility rate, indicating the number of patients that were scheduled for ALPPS and completed the procedure, was reported to be between 97 and 100% [18, 19]. Taken together, ALPPS is the method with the most effective and fastest increase of FLR associated with the highest feasibility rate to complete the strategy which is currently available.

The obvious fear associated with strategies for increasing the FLR in patients with CRLM is the induction of tumor growth and enhancement of metastatic spread during the waiting period. DeGraaf and coworkers suggested three mechanisms that might enhance tumor growth upon PVE: induction of cytokine/growth factor secretion, changes of hepatic blood flow (arterialization), and a modified cellular host response [20]. Data concerning the impact of PVE on tumor proliferation and subsequently on overall (OS) and disease-free survival (DFS) are controversial [13, 16, 21–24]. Regarding ALPPS, almost no data are available about its effects on the tumor. Only Tanaka and coworkers compared patients with TSH/PVE and ALPPS regarding Ki67 expression index in the tumor (at first and second stages) showing a significant induction of Ki67 in TSH/PVE but not in ALPPS [25]. In contrast, Fukami described an increase in Ki67 labeling index in a single patient after ALPPS [26]. The aim of our study was to compare patients that underwent standard liver resection (one-stage) with those that underwent ALPPS regarding changes in tumor cell proliferation, apoptosis, and vascularization.

## Methods

Patients undergoing ALPPS (*n =* 6) were 1:2 matched with patients after standard (one-stage) liver resection. One matched patient after standard liver resection had to be excluded from further analyses since no tumor material was available for this study. Therefore, only 11 patients after standard liver resection were included into the analyses. Matching was performed regarding size, number (both based on the postoperative pathologic reports), and timing of appearance of the metastases (syn-/metachronous), preoperative chemotherapy, localization, TNM stage, and grading of the primary tumor, as well as patient characteristics (age, gender). In case of neoadjuvant therapy, size of the biggest lesion was measured at the time of initial staging before chemotherapy and upon re-staging just before resection from CT scans. To determine changes in the size of the lesions during ALPPS procedure, size of the biggest metastasis was determined from CT scans just before the first and prior to the second stage. All patients underwent resection at the Department of Surgery, University Hospital Regensburg between 2006 and 2011. Informed consent was obtained from all patients prior to surgery. Data were collected retrospectively. The study was approved by the local IRB at the University Hospital Regensburg (No. 15-101-0039).

### Histological analysis

The largest resected metastasis based on the postoperative pathological report of ALPPS group was compared to the largest in the standard liver resection group. Tissue samples of CRLM were fixed in formalin and paraffin embedded prior to cutting them at 3 μm. SuperFrost Plus slides (Thermo Scientific, Massachusetts, USA) were used to perform immunohistochemistry and TUNEL staining.

### Immunhistochemistry

The Roche Ventana BenchMark Ultra staining system and the ultraView DAB detection kit were used for all immunostainings. After warming the slides up to 75 °C, deparaffinizing and pretreating them (not for αSMA), the staining kit was applied. Having added 3% H_2_O_2_, the slides were incubated with the primary antibody. To visualize the bound structures, the secondary antibody, diaminobenzidine (DAB), H_2_O_2_, and copper were applied. Having counterstained the sections and washed them twice, they were dehydrated through increasing alcohol concentration and covered with Entellan® (Merck Millipore, Darmstadt, Germany).

Tumor specimens were stained for Mib-1/Ki67 (proliferation, mouse anti-human monoclonal Ki67 antibody, DAKO, 1:100), cleaved caspase-3 (apoptosis, rabbit polyclonal antibody, Cell Signaling, 1:100), CD31 (vascularization, mouse anti-human monoclonal antibody, DAKO, 1:20), and αSMA (smooth muscle actin, pericytes, mouse anti-human monoclonal, Chemicon, 1:500). Measurements of the tumor slide were performed in four random fields at ×40 magnification at the tumor invasive front. Ki67-positive and caspase-3-positive tumor cells were counted per high-power field (hpf), and averages were calculated. CD31-positive stained vessels and αSMA-positive areas were measured using ImageJ software and expressed as pixels per hpf.

### TUNEL assay

To detect DNA fragmentation and consequently apoptotic cells in tumor specimens, terminal deoxynucleotidyl transferase-mediated dUTP nick end labeling assay (TUNEL; DeadEnd™ Colorimetric Tunel System, Promega) was used according to the manufacturer’s instructions. In brief, formalin-fixed and paraffin-embedded slides (3 μm) were deparaffinized by Xylol, hydrogenated through a graded ethanol series, 0.85% NaCl, and PBS buffer, and fixed with 4% paraformaldehyde. After covering the tissues with protein kinase K, they were incubated with the reaction mixture containing terminal deoxynucleotidyl transferase, equilibration buffer, and biotinylated nucleotide mix for 1 h at 37 °C to label 3ʹOH DNA ends. Streptavidin- and DAB-bound biotin was quantified, and nuclei were stained dark brown. Slides were counterstained with hemalaun (Merck Millipore, Darmstadt, Germany) and covered with aquatex (Merck Millipore, Darmstadt, Germany). DNA fragmentation was detected by selecting four fields at ×40 magnification in each tumor section at the invasive front and counting positive stained nuclei. Data were expressed as the number of apoptotic cells per high-power field.

### Statistical analyses

To assess any significance between both groups, Student’s *t* test and Fisher’s exact test were used. For both tests, a *p* value <0.05 was considered to be statistically significant. Results were expressed as the mean ±SD.

## Results

Characteristics of the six patients with ALPPS and 11 patients with standard liver resection regarding primary tumors and liver metastases are summarized in Table 1. No significant difference was observed between both groups. Patients without preoperative therapy underwent liver resection within 2–3 weeks after diagnosis. In case of neoadjuvant therapy (*n =* 6 patients), liver resection was performed 4–6 months after the initial diagnosis. All patients in the ALPPS group underwent extended right hepatectomy (+/− Seg. I, *n =* 3 each). Median time between step 1 and step 2 was 10 days (range 8–12). Based on the CT scans, the size of the largest lesion before the first step of ALPPS was 6.22 cm (±1.30) and before the second step of ALPPS was 6.43 (±1.41) (*p =* 0.793). Standard liver resection included six patients with multiple atypical resections, one right and one left hemihepatectomy, one left hemihepatectomy with atypical resection on the right side, and two extended right hepatectomies.

**Table 1:** Characteristics of patients, primary tumors, and metastases

	ALPPS (*n =* 6)	Standard liver resection (*n =* 11)	*p* value
Age	67.1 ± 3.48	64.1 ± 3.45	0.522
Male/female	4/2	5/6	0.620
T stage			
1	0	0	1.0
2	1	1	1.0
3	3	7	0.644
4	2	3	1.0
N stage			
0	3	4	0.644
1	2	3	1.0
2	1	4	0.6
Grading			
1	0	0	1.0
2	4	8	1.0
3	2	3	1.0
Number of metastases	3.12 ± 1.28	2.56 ± 0.55	0.607
Size of metastases	6.80 ± 1.46	4.06 ± 0.55	0.126
Syn/meta	3/3	6/5	1.0
Preoperative chemotherapy	2	4	1.0
Tumor size at the time of diagnosis (before preoperative chemotherapy)^a^	4.90 ± 1.60	2.95 ± 1.20	0.161
Tumor size before resection (after preoperative chemotherapy)^a^	2.85 ± 0.15	2.35 ± 1.01	0.547

^a^Based on CT scan

### Tumor cell proliferation

**Fig. 1:**
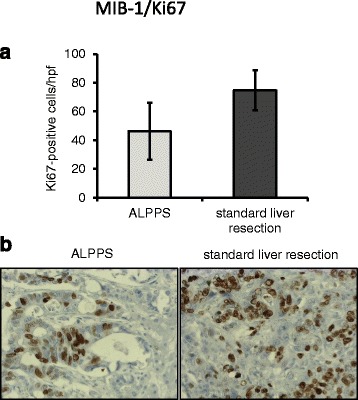
Tumor cell proliferation in ALPPS vs. standard liver resection. **a** No significant difference was found regarding Ki67-positive cells. **b** Examples for Ki67 staining in ALPPS (*left side*) and standard liver resection (*right side*)

Tumor cell proliferation was determined by Ki67 staining. Results showed a lower number of Ki67-positive tumor cells in the ALPPS group (46.08 ± 19.88 cells/hpf) than after standard liver resection (74.73 ± 13.89 cells/hpf; Fig. 1). However, this did not reach statistical significance (*p =* 0.244).

### Apoptosis

**Fig. 2:**
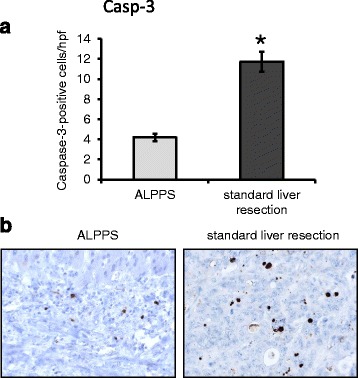
Caspase-3 staining in ALPPS vs. standard liver resection. **a** Significantly less caspase-3-positive cells were detected after ALPPS (*p < 0.001). **b** Examples for caspase-3 staining in ALPPS (*left side*) and standard liver resection (*right side*)

**Fig. 3:**
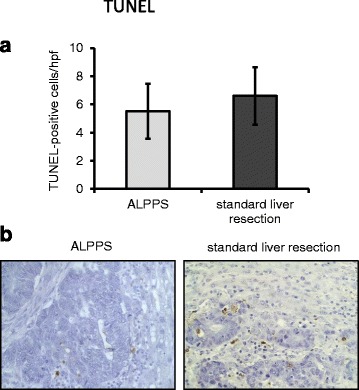
TUNEL-positive cells in ALPPS vs. standard liver resection. **a** No significant difference was found regarding TUNEL-positive cells. **b** Examples for TUNEL staining in ALPPS (*left side*) and standard liver resection (*right side*)

Caspase-3 staining revealed significantly fewer apoptotic cells after ALPPS than after standard liver resection (11.73 ± 1 cells/hpf vs. 4.19 ± 0.38 cells/hpf; *p <* 0.001; Fig. 2). To confirm this finding, TUNEL staining was performed. Although the number of TUNEL-positive cells was also decreased upon ALPPS, results did not show a significant difference between ALPPS and standard liver resection (5.52 ± 1.94 cells/hpf vs. 6.6 ± 2.04 cells/hpf; *p =* 0.734; Fig. 3).

### Vascularization

**Fig. 4:**
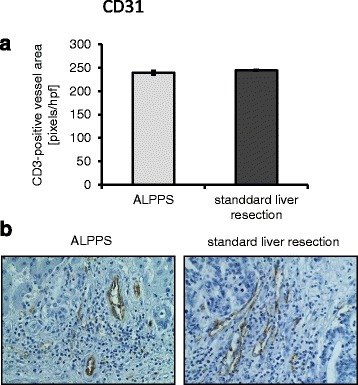
CD31 vessel area in ALPPS vs. standard liver resection. **a** No significant difference was found regarding CD31 vessel area. **b** Examples for CD31 staining in ALPPS (*left side*) and standard liver resection (*right side*)

**Fig. 5:**
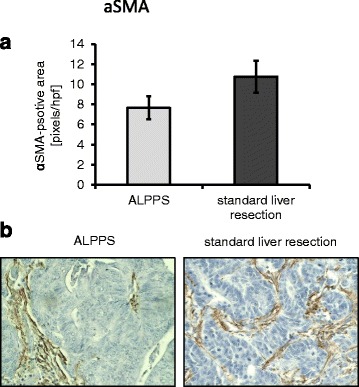
αSMA positive area in ALPPS vs. standard liver resection. **a** No significant difference was found regarding αSMA positive area. **b** Examples for αSMA staining in ALPPS (left side) and standard liver resection (*right side*)

Vascularization was determined by a CD31 vessel area. Results showed no significant difference between the ALPPS group (239.46 ± 4.08 pixels/hpf) compared to patients that underwent standard liver resection (244.43 ± 1.16 pixels/hpf; *p =* 0.149; Fig. 4). Since pericytes have been implicated in tumor growth and vascularization, αSMA was assessed. Again, no significant difference between ALPPS and standard liver resection was found (7.65 ± 1.15 pixels/hpf vs. 10.75 ± 1.6 pixels/hpf; *p =* 0.205; Fig. 5).

## Discussion

The present study provides evidence that ALPPS is not associated with parameters characteristic of increased tumor growth at the time point of final resection of metastases. Results show no appreciable difference in tumor cell proliferation (Ki67), apoptosis (TUNEL), vascularization (CD31), or pericyte coverage (αSMA) when ALPPS specimens were compared to standard liver resection.

Several strategies to increase the FLR in patients with advanced CRLM or primary liver cancer are in use. Most frequently, PVE of the contralateral liver lobe is performed. The effects of strategies to increase the FLR on tumor cell proliferation have been addressed in several studies. Animal experiments suggest that partial hepatectomy increases the growth of colorectal liver metastases in liver remnants [27]. With regard to portal vein occlusion, a rat model by Maggiori et al. showed that the tumor volume in the embolized liver lobe was smaller compared to non-embolized controls, whereas the opposite effect was observed on the contralateral side [28]. In contrast, Hoekstra et al. found an increase in tumor growth rate in the embolized liver lobe after portal vein occlusion in a rabbit model [29]. The same group confirmed the latter findings in a clinical setting when comparing 28 patients after PVE with 30 patients without preoperative PVE [22]. Pamecha and coworkers showed a higher mitotic rate and a significant increase in Ki67 proliferation index after PVE, but no differences regarding necrosis or apoptosis when comparing samples from patients with preoperative PVE to those without PVE [30]. With regard to ALPPS, an increase in Ki67 labeling index from 60 to 80% between step 1 and step 2 of the procedure was reported in a single patient with CRLM [26]. Very recently, Tanaka et al. compared 10 patients after ALPPS with 47 patients after “classical” two-stage hepatectomy, including PVE/PVL. Results show no difference in Ki67 expression between both groups after the first step, but higher Ki67 expression was observed after the second operation with the classical two-stage procedure [25]. Findings from our present study go along with those of Tanaka and coworkers, since we did not detect any difference between ALPPS and standard liver resection with regard to Ki67 expression, although our control group (standard (one-stage) liver resection) is different. Moreover, we did not detect a difference regarding apoptosis in our study upon ALPPS which is similar to the results from Pamecha et al. upon PVE [30]. Finally, we determined the effects of ALPPS on metastases vascularization (CD31) and pericyte coverage (αSMA). To our knowledge, this is the first time that this was addressed in this setting. Nonetheless, the issue of vascularization is of particular importance since Schadde *et al.* very recently described an increase of local hypoxia and a consecutive increase in HIF-1α (hypoxia-inducible factor-1α) expression in an experimental rat model after ALPPS [31]. Since the hypoxia-driven increase in HIF-1α is the main stimulus for VEGF-A (vascular endothelial growth factor-A)-induced angiogenesis, one might have expected an effect on vascularization. However, neither regarding vascularization (CD31) nor regarding pericyte coverage (αSMA) a difference was detectable. Taken together, our results indicate that ALPPS does not have a significant impact on the tumor itself.

The obvious fear is that strategies increasing growth of the liver remnant might negatively impact on OS or DFS. With regard to PVE, clinical data on long-term survival is controversial. A study by Hoekstra et al. reported 3-year overall survival of 26% after PVE versus 77% without preoperative embolization [22]. In contrast, Ardito and coworkers found no difference in OS, DFS, or liver-specific DFS when comparing 20 patients after PVE with the same number of patients without PVE before right or extended right hepatectomy [21]. Moreover, a study by Simoneau et al. showed no difference regarding OS even upon tumor progression after PVE. However, patients with tumor progression had significantly shorter DFS [23]. Recently, a systematic review and meta-analysis by Giglio and coworkers concluded that PVE has no negative impact on hepatic recurrence and OS [32]. With respect to ALPPS, data are even more limited. Oldhafer and colleagues reported early recurrence after a median of 8 months in six of seven patients that underwent ALPPS for CRLM [33]. We have recently published data from our experience showing a median DFS of 18.7 months and 3-year OS of 64% after ALPPS for CRLM [18]. Schadde et al. reported 1- and 2-year DFS of 59 and 41%, respectively, with a median DFS of 14 months from the ALPPS registry [34]; 1- and 2-year OS in this study was 76 and 62%, respectively. Finally, Alvarez et al. reported a DFS of 67% after 1 year and 40% after 2 years, although only 19 of 30 patients in this study suffered from CRLM [35]. The high and early recurrence rates after ALPPS compared to PVE might be explained by the fact that a significant number of patients after PVE do not undergo liver resection due to disease progression or insufficient growth of the liver remnant [36]. Therefore, PVE is sometimes considered to be a better selection method for patients undergoing extensive liver resection for CRLM. However, one has to keep in mind that even after standard liver resection with curative intent, recurrence rates up to 70% have been reported [37]. In summary, much more data is available concerning long-term OS and DFS after PVE than after ALPPS for CRLM, but the impact of both procedures on oncologic survival recurrence is still not clear so far.

Our study harbors some limitations. First, the number of patients included is limited. One reason for that is the novelty of the ALPPS approach with limited patients being treated in this way to date. Second, the control group of our population is very heterogeneous. Optimal control for assessing effects of ALPPS on tumor cells would have been a biopsy from the metastases upon the first stage of the procedure. However, to avoid any harm to the patient, e.g., by biopsy-driven tumor spread, we did not do this. Third, we could not provide data on long-term OS and DFS. This owes to the fact that one of the six patients in the ALPPS group died in the early postoperative phase, and therefore, oncologic survival analyses were not reasonable. The latter even shows that ALPPS is a procedure with relevant morbidity and mortality that should only be performed in patients with advanced liver metastases that are otherwise not eligible for surgery.

## Conclusions

The results of our study showed no difference regarding proliferation, apoptosis, and vascularization in CRLM when comparing matched patients that underwent ALPPS with those that underwent standard (one-step) liver resection. The study, therefore, increases the body of evidence for the use of ALPPS in selected patients with otherwise unresectable CRLM.

## Abbreviations

ALPPS: Associating liver partition and portal vein ligation for staged hepatectomy
CRLM: Colorectal liver metastases
DFS: Disease-free survival
FLR: Future liver remnant
HIF-1α: Hypoxia-inducible factor-1α
hpf: High-power field
IRB: Institutional review board
ISS: In situ split
OS: Overall survival
PHLF: Posthepatectomy liver failure
PVE: Portal vein embolization
TSH: Two-stage hepatectomy
TUNEL: TdT-mediated dUTP-biotin nick end labeling
VEGF-A: Vascular endothelial growth factor-A
αSMA: α-Smooth muscle actin
